# Elevated Expression of Calpain-4 Predicts Poor Prognosis in Patients with Gastric Cancer after Gastrectomy

**DOI:** 10.3390/ijms17101612

**Published:** 2016-09-27

**Authors:** Peike Peng, Lingqiang Min, Shushu Song, Junjie Zhao, Lili Li, Caiting Yang, Miaomiao Shao, Mingming Zhang, Hao Wu, Jie Zhang, Can Li, Xuefei Wang, Hongshan Wang, Jing Qin, Yuanyuan Ruan, Jianxin Gu

**Affiliations:** 1 Department of Biochemistry and Molecular Biology, School of Basic Medical Sciences, Fudan University, Shanghai 200032, China; 14111010028@fudan.edu.cn (P.P.); songshus@126.com (S.S.); 13111010011@fudan.edu.cn (L.L.); 15111010021@fudan.edu.cn (C.Y.); 12111010008@fudan.edu.cn (M.S.); 14111010031@fudan.edu.cn (M.Z.); 15211010029@fudan.edu.cn (H.W.); 14111010002@fudan.edu.cn (C.L.); gjxjxg@126.com (J.G.); 2 Department of General Surgery, Zhongshan Hospital, Fudan University, Shanghai 200032, China; minlingqiang@163.com (L.M.); 14111210064@fudan.edu.cn (J.Z.); wang.xuefei@zs-hospital.sh.cn (X.W.); 3 Institute of Biomedical Science, Fudan University, Shanghai 200032, China; 15211510023@fudan.edu.cn

**Keywords:** gastric cancer, calpain-4, prognosis, biomarker, overall survival, nomogram

## Abstract

Calpain-4 belongs to the calpain family of calcium-dependent cysteine proteases, and functions as a small regulatory subunit of the calpains. Recent evidence indicates that calpain-4 plays critical roles in tumor migration and invasion. However, the roles of calpain-4 in gastric tumorigenesis remain poorly understood. Herein, we examined calpain-4 expression by immunohistochemical staining on tissue microarrays containing tumor samples of 174 gastric cancer patients between 2004 and 2008 at a single center. The Kaplan-Meier method was used to compare survival curves, and expression levels were correlated to clinicopathological factors and overall survival. Our data demonstrated that calpain-4 was generally increased in gastric cancer cell lines and primary tumor tissues. High expression of calpain-4 was positively associated with vessel invasion, lymph node metastasis, and advanced TNM (Tumor Node Metastasis) stage. Multivariate analysis identified calpain-4 as an independent prognostic factor for poor prognosis. A predictive nomogram integrating calpain-4 expression with other independent prognosticators was constructed, which generated a better prognostic value for overall survival of gastric cancer patients than a TNM staging system. In conclusion, calpain-4 could be regarded as a potential prognosis indicator for clinical outcomes in gastric cancer.

## 1. Introduction

Gastric cancer remains the fifth most common malignancy and the third leading cause of cancer-associated death worldwide, especially in Asia [1]. Due to the lack of specific symptoms at the early stage, over 80% of patients with gastric cancer are diagnosed at an advanced and unsuitable stage for surgical resection, which is the major reason for a poor prognosis [2]. The clinically used prognostic model for outcomes of gastric cancer patients mainly relies on a tumor cell-derived TNM (Tumor Node Metastasis) stage [3]. Nevertheless, a growing body of evidence has suggested that patients with the same TNM stage might have vastly different prognoses due to the heterogeneity of tumors [4,5]. Hence, useful prognostic biomarkers or underlying mechanisms are urgently needed to refine risk stratification for prognosis of patients with gastric cancer.

Calpains are a family of intracellular Ca^2+^-regulated cysteine proteases, evolutionarily well-conserved from bacteria to mammals [6]. The archetypal members of calpain family, calpain-1 (μ-form) and calpain-2 (m-form), are ubiquitously expressed, while the other members are tissue-specific cysteine proteases that proteolyze different kinds of substrates, leading to their degradation or functional modulation [6]. Calpain activity has been implicated in several fundamental physiological processes, including cytoskeletal remodeling, migration, proliferation, cell cycle control, survival and apoptosis [7]. Aberrant calpain expression is observed in numerous pathological conditions, including myocardial infarction, neurodegeneration, multiple sclerosis and cancer [8]. Calpain-4 (calpain small subunit 1, CAPNS1) is a small regulatory subunit of the calpains which plays a critical role in maintaining calpain stability and activity [9]. Lack of calpain-4 affects the function of μ-calpain and m-calpain and leads to lethality in the early embryonic stage [9,10]. A recent study indicates that calpain-4 could regulate the organization of the actin cytoskeleton during cell migration [11]. Overexpression of calpain-4 is observed in several types of tumors, such as hepatocellular carcinoma, intrahepatic cholangiocarcinoma, and clear cell renal cell carcinoma, and could be regarded as a predictor of poor prognosis [12,13,14,15,16,17]. Nevertheless, the roles of calpain-4 in gastric carcinoma remain poorly understood.

The aim of the present study was to investigate the expression of calpain-4 in gastric cancer specimens and explore its associations with clinicopathological factors and prognosis. We also evaluated whether integration of intratumoral calpain-4 expression could generate a predictive nomogram to refine the risk stratification system for prognosis of patients with gastric cancer.

## 2. Results

### 2.1. Expression of Calpain-4 Was High in Gastric Cancer

**Figure 1:**
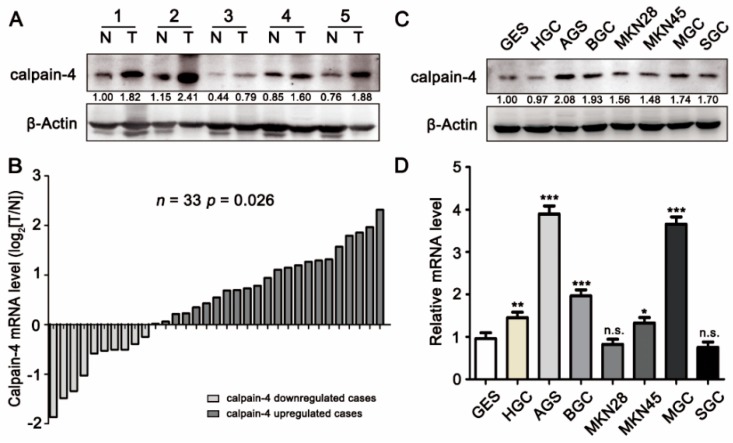
Calpain-4 expression is up-regulated in gastric cancer tissues and cell lines. (**A**) The protein levels of calpain-4 in 5 representative paired gastric cancer and adjacent normal tissues were detected by Western blot analysis. N, adjacent normal tissues; T, matched gastric cancer tissues; (**B**) the mRNA levels of calpain-4 in 33 cases of gastric cancer and paired adjacent normal tissues were determined by real-time PCR. Gray represents samples with calpain-4 down-regulation, while black represents samples with calpain-4 up-regulation; (**C**,**D**) the protein and mRNA levels of calpain-4 in GES-1 and 7 gastric cancer cell lines were examined by western blot (**C**) and real-time PCR (**D**) analysis. In (**D**), the statistics were made by comparing with GES-1 group, respectively. The gels have been run under the same experimental conditions. * *p* < 0.05, ** *p* < 0.01, *** *p* < 0.001, n.s., no significance.

To understand whether calpain-4 was involved in gastric carcinogenesis, we first examined the protein expression of calpain-4 in gastric cancer tissues compared with matched adjacent normal gastric mucosa by Western blot (Figure 1A). Results revealed that calpain-4 protein levels were up-regulated in gastric cancer. To confirm this conclusion, we next investigated the mRNA levels of calpain-4 in 33 paired gastric cancer samples, and the results showed that the mRNA expression of calpain-4 was higher in tumors (Figure 1B). We also examined the expression of calpain-4 in normalized gastric mucosa cell line GES-1 and various gastric cancer cell lines (HGC-27, AGS, BGC-823, MKN-28, MKN-45, MGC80-3, and SGC-7901). The results demonstrated that all gastric cancer cell lines except for HGC-27 displayed higher protein expression of calpain-4 than the GES-1 cell line (Figure 1C). A real-time PCR assay also revealed that 5 (HGC-27, AGS, BGC-823, MKN45 and MGC80-3) out of the 7 gastric cancer cell lines showed higher calpain-4 mRNA levels than GES-1 cells (Figure 1D). These results suggested that calpain-4 expression is increased in gastric cancer.

### 2.2. Immunohistochemical Findings and Association between Calpain-4 Expression and Clinicopathologic Characteristics in Patients with Gastric Cancer

**Figure 2:**
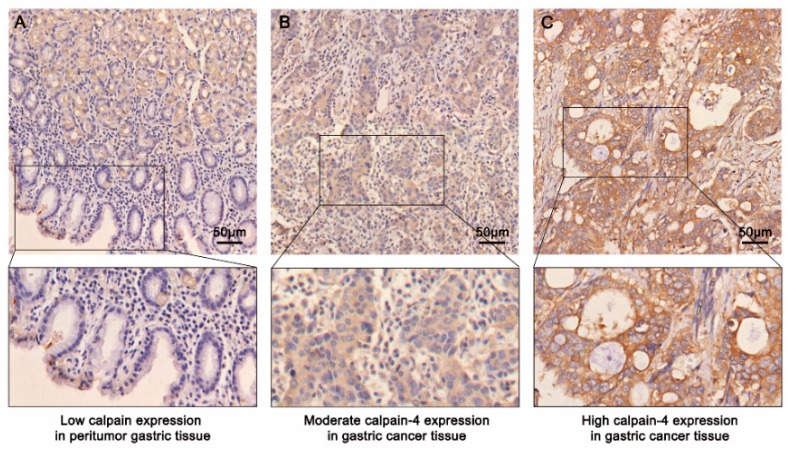
Representative images of tissue microarray stained for calpain-4 and its regional magnification in gastric cancer sections and normal gastric mucosa. Normal gastric epithelium showed low calpain-4 expression (**A**); while gastric cancer tissues showed moderate to high calpain-4 expression (**B**,**C**). Scale bar = 50 μm.

To further evaluate the protein level of calpain-4 in gastric cancer tissues, we detected the expression of calpain-4 by immunohistochemical staining analysis in 174 patients with gastric cancer. As shown in Figure 2, calpain-4 staining was mainly on the cytoplasm of tumor cells. The calpain-4 staining varied greatly among different specimens. The relationships between clinical pathological characteristics and caplain-4 expression are shown in Table 1. High calpain-4 expression was positively correlated with vessel invasion (*p* = 0.018), lymph node metastasis (*p* < 0.001) and advanced TNM stage (*p* = 0.006). Collectively, these observations suggest that increased calpain-4 expression in tumor cells is associated with the progression of gastric cancer.

### 2.3. Association between Calpain-4 Expression and Prognosis of Patients with Gastric Cancer

**Figure 3:**
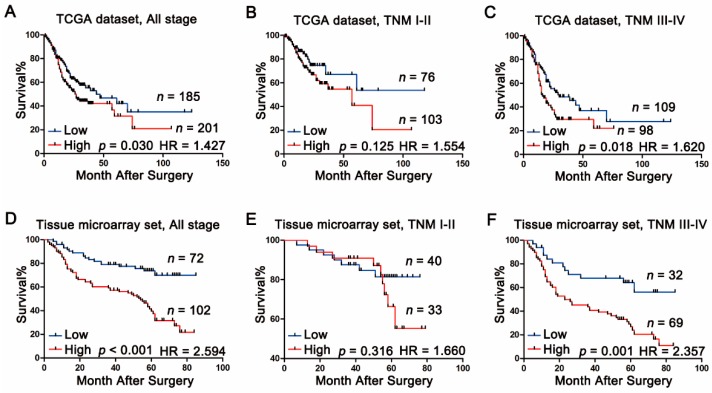
The predictive value of calpain-4 expression in patients with gastric cancer. Kaplan-Meier analysis for overall survival of patients with gastric cancer according to the calpain-4 expression in TCGA dataset (**A**–**C**); and in our tissue microarray set (**D**–**F**). HR, Hazard ratio; *p*-value was calculated by log-rank test.

To further explore the prognostic value of calpain-4 expression in gastric cancer, Kaplan-Meier survival analysis was also applied to compare overall survival according to calpain-4 expression. In both the TCGA dataset and our tissue microarray set, Kaplan-Meier survival analysis indicated that gastric cancer patients with high calpain-4 expression had a worse prognosis than those patients with low calpain-4 expression (Figure 3A,D). To further investigate whether calpain-4 expression could stratify patients with different TNM stages, we divided the patients with TNM I–II into the early-stage group and TNM III–IV into the advanced-stage group. In both datasets, high expression of calpain-4 was associated with poor overall survival in patients of advanced-stage groups (Figure 3C,F), while no significant correlation was found between calpain-4 expression and overall survival in patients of early-stage groups (Figure 3B,E). Further analysis indicated that high calpain-4 expression was only correlated with vessel invasion and was not a prognostic risk factor in the early-stage group. These observations demonstrated that high expression of calpain-4 in tumor tissues might indicate unfavorable survival in advanced gastric cancer rather than all gastric cancer.

**Figure 4:**
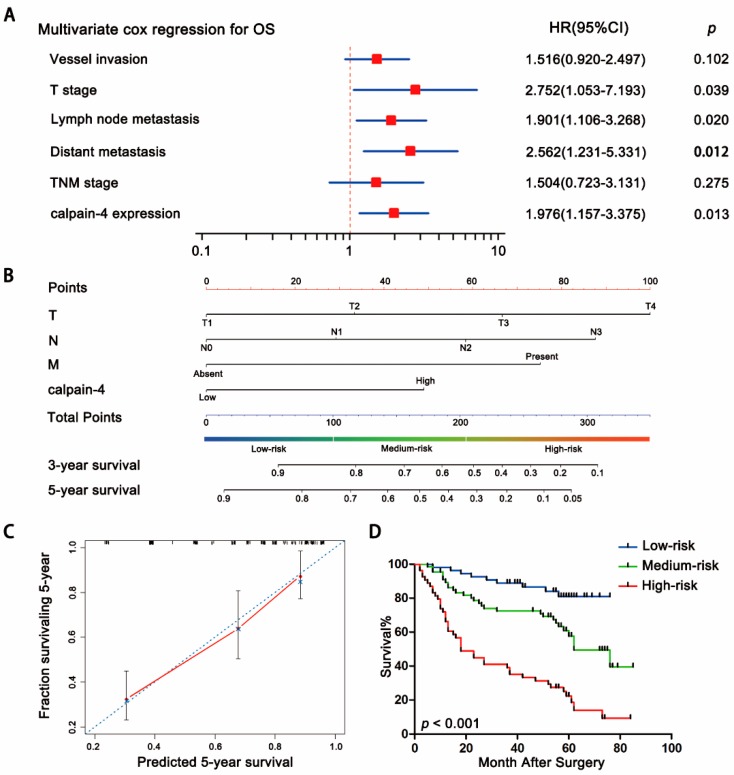
Nomogram for predicting overall survival in patients with gastric cancer. (**A**) Multivariate Cox regression analysis identified independent prognostic factors for overall survival; (**B**) nomogram for predicting clinical outcomes was generated, integrating calpain-4 expression (low/high) with T stage (T; T1/T2/T3/T4), lymph node metastasis (N; N0/N1/N2/N3), and distant metastasis (M; Absent/Present); (**C**) calibration plot for predicting survival at 5 years. The nomogram showed good performance with the ideal model; (**D**) the patients were divided into 3 groups according to the total points calculated by the nomogram, followed by Kaplan-Meier analysis for overall survival of patients in each group. *p*-value was calculated by log-rank test.

Univariate Cox analysis was used to evaluate the prognostic value of clinicopathological factors for overall survival. Vessel invasion (*p* = 0.033), T stage (*p* < 0.001), lymph node metastasis (*p* < 0.001), distant metastasis (*p* < 0.001), TNM stage (*p* < 0.001), and calpain-4 expression (*p* < 0.001) were found to be risk factors for survival in patients with gastric cancer (Table 2). Further analysis with multivariate Cox regression identified T stage (*p* = 0.039), lymph node metastasis (*p* = 0.020), distant metastasis (*p* = 0.012) and calpain-4 expression (*p* = 0.013) as independent risk factors for gastric cancer patients (Figure 4A). These data indicated that high expression of calpain-4 might be an independent factor that predicts poor prognosis in patients with gastric cancer.

### 2.4. Predictive Nomogram for Overall Survival of Patients with Gastric Cancer

In order to establish a quantitative method to better stratify patients with different clinicopathological features, we constructed a prognostic nomogram that integrated the independent factors for overall survival selected by multivariate analysis, including T stage, lymph node metastasis, distant metastasis and calpain-4 expression. In this nomogram, a higher total point indicated a poorer prognosis. The total point was calculated by adding the score of T stage (0 for “T1”, 33 for “T2”, 67 for “T3” or 100 for “T4”), lymph node metastasis (0 for “N0”, 29 for “N1”, 58 for “N2” or 88 for “N3”), distant metastasis (0 for “Absent” or 75 for “Present”) and calpain-4 expression (0 for “Low” or 49 for “High”) for each patient (Figure 4B). The calibration curve for predicted 5-year overall survival showed that the nomogram performed well with the ideal prediction model (Figure 4C). Furthermore, we divided patients into 3 groups according to the total points calculated by the nomogram: the high-risk (>75th percentile of the group), medium-risk (25th–75th percentile) and low-risk (<25th percentile) groups. Overall survival in each group was found to increase following the trend from high- to low-risk groups, which demonstrated that scoring with the nomogram effectively discriminated the risk of postoperative gastric cancer development (Figure 4D). The AIC was 709.79 when estimated according to TNM stage alone, and it decreased to 700.72 when estimated in the generated nomogram. The Harrell’s concordance index (*C*-index) for the generated nomogram was 0.742 (95% CI, 0.691–0.792), higher than 0.693 (95% CI, 0.648–0.739) of TNM alone, indicating that the nomogram showed a better value in predicting the overall survival of gastric cancer patients.

### 2.5. Knockdown of Calpain-4 Expression Reduced the Invasiveness of Gastric Cacner Cell in Vitro

**Figure 5:**
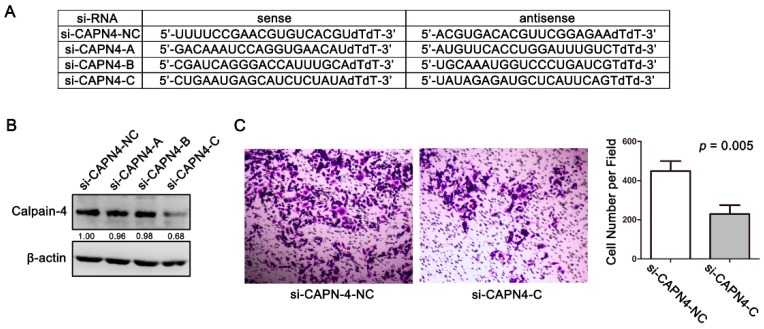
Knockdown of calpain-4 expression reduced the invasiveness of gastric cancer cells in vitro. (**A**) The sequences of calpain-4 specific siRNA. NC, negative control; (**B**) knockdown of calpain-4 expression in AGS cells; (**C**) knockdown of CAPN4 inhibited the invasion of AGS cells, original magnification: 100×.

We next examined the role of calpain-4 in the invasion of gastric cancer cells. Three pairs of CAPN4-siRNA were used to knock down calpain-4 expression in AGS cells (Figure 5A). Western blot analysis showed that si-CAPN4-C could effectively silence the expression of calpain-4 (Figure 5B). After the knockdown of CAPN4, we investigated the invasive potential of AGS cells by transwell matrigel invasion assays, and found that calpain-4 inhibition could reduce the invasiveness of gastric cancer cells in vitro (Figure 5C).

## 3. Discussion

As is well known, gastric cancer is a highly heterogeneous disease with poor clinical outcome. To provide a predictive model for patients with gastric cancer, traditional tumor-node-metastasis (TNM) staging system and the Lauren classification have been commonly used in clinical practice [3,18]. However, these predictive models still have a limit in their ability to discriminate a subset of patients when referring to the molecular heterogeneity of gastric cancer [5]. Therefore, identifying molecules with tumorigenic properties is helpful for understanding the tumor progression of gastric cancer. Recently, the Asian Cancer Research Group (ACRG) applied gene expression profiling and defined four distinct gastric cancer molecular subtypes that are associated with distinct genomic alterations, survival outcome and recurrence patterns after surgery [19], which shows the prognostic value of gastric cancer by using molecular approaches. To our knowledge, this study was the first to demonstrate an association between high calpain-4 expression and poor prognosis in gastric cancer patients following surgery. Our work suggested that calpain-4 might play an important role in the development of gastric cancer. Therefore, the molecular mechanism and potential functions of calpain-4 in gastric cancer need further investigation.

Calpains are a large superfamily of intracellular Ca^2+^-dependent non-lysosomal cysteine proteases, which can be divided into typical and atypical members according to their domain structures [7,20]. Ubiquitous μ- and m-calpains, defined by the calcium concentration required for their proteolytic activities [7,21], could form a heterodimer with the small 28 kDa regulatory subunit (calpain-4), respectively [22]. As a regulatory subunit of calpains, calpain-4 is ubiquitously expressed in tissues and plays a crucial role in various biological functions. Absence of a functional calpain-4 would abolish the activity of the classical μ- and m-calpains, and severely affect the proliferation and differentiation of cells of the osteoblast lineage [9,10]. In addition to its traditional role as the regulatory subunit of calpains, calpain-4 also functions as a regulatory binding partner of several proteins. A previous study indicates that calpain-4 combines with PTH1R (parathyroid hormone-related peptide receptor) through an N-terminal portion of the PTH1R’s intracellular tail, leading to C-terminal shortening of PTH1R and enhanced PTH1R downstream signaling [23]. Calpain-4 also binds to the αPIX (PAK-interacting exchange factor) via the SH3-DH-PH triple domain, and regulates the activity of αPIX to promote integrin-mediated signaling and cell spreading [24].

In tumorigenesis, the prognostic and therapeutic value of calpain-4 have been illustrated in hepatocellular carcinoma, intrahepatic cholangiocarcinoma, clear cell renal cell carcinoma, non-small cell lung cancer, nasopharyngeal carcinoma and glioma [12,13,14,15,16,17]. High calpain-4 expression is found to be positively correlated with tumor invasion and metastasis, and predicts poor prognosis in these types of malignancies. It is well recognized that calpain-4 is a small regulatory subunit of the calpain which plays a critical role in maintaining the stability of calpain family, including calpain-1, calpain-2 and calpain-9. Nevertheless, our previous data indicated that “the expression of calpain-9 was down-regulated in gastric cancer, and high calpain-9 expression oppositely predicted favorable prognosis in patient with gastric cancer” [25]. Though the mechanisms underlying the decreased expression of calpain-9 in gastric cancer remains to be defined, these data suggest that other pathways rather than calpain-9 may be involved in the tumorigenic effect of calpain-4. For example, recent research suggests the role of calpain-1 in proteolytic cleavage of β-catenin which leads to aberrant stabilization of the protein and increased tumorigenic potential in cancer cells [26]. Overexpression and enhanced activity of calpain-2 also induces an increase in the fragmental cleavage of AR and FlnA, which may contribute to the development of an aggressive phenotype of prostate cancer [27]. Recent research suggest that calpain-4 promotes cell spreading and migration by disassembling focal adhesions in the trailing edge of cells [28]. In addition, calpain-4 overexpression can activate FAK (Focal adhesion kinase)-Src signaling pathways and up-regulate MMP2 expression to induce tumor metastasis [14]. Similar results were also found in our study that high level expression of calpain-4 was associated with vessel invasion, lymphatic node metastasis and TNM stage in gastric cancer. Furthermore, high calpain-4 expression was correlated with poor overall survival in patients at the TNM III + IV stage, suggesting that calpain-4 might play a significant role in gastric cancer at advanced stage. How calpain-4 regulates the invasive activities in gastric cancer may also need further investigation.

## 4. Materials and Methods

### 4.1. Primary Gastric Cancer Samples

All of the methods were performed in accordance with the approved guidelines. Primary tumor specimens of gastric cancer were obtained from 174 gastric cancer patients who underwent gastrectomy without preoperative treatment in the Department of General Surgery, Zhongshan Hospital (Fudan University, Shanghai, China), between 2004 and 2008. Relevant clinicopathological features of these patients including age, gender, tumor location, tumor differentiation, Lauren classification, vessel invasion and TNM stage were collected retrospectively. Tumor stage and tumor differentiation grade were reclassified according to the seventh American Joint Committee on Cancer TNM classification by two independent gastroenterology pathologists. The median age of this cohort was 61 years (range 31–86 years), and 64.4% of the cohort were male. Intestinal, mixture and diffuse histologic subtypes constituted 62.6%, 6.9% and 30.5% of cases, respectively. Overall survival was defined as the interval between the date of surgery and the date of death or last visit. Frozen gastric cancer and matched adjacent normal mucosa tissues were also obtained from the Department of General Surgery, Zhongshan Hospital (Fudan University, Shanghai, China) in 2014. Adjacent normal mucosa tissues were obtained from sites that were >60 mm away from primary lesions. The study was approved by the Research Ethics Committee of Zhongshan Hospital (2015-006, May 2015), and informed consent was obtained from every patient.

### 4.2. Cell Lines

Seven gastric cancer cell lines (HGC-27, AGS, BGC-823, MKN-28, MKN-45, MGC80-3, and SGC-7901) and a gastric mucosal cell line (GES-1) were obtained from the Cell Bank of Type Culture Collection of Chinese Academy of Sciences (Shanghai, China). HGC27, MKN-28, and MKN-45 cells were cultured in Dulbecco’s Minimum Essential Medium and the other cell lines were cultured in RPMI-1640 Medium, supplemented with 10% fetal bovine serum at 37 °C in a humidified atmosphere containing 5% CO_2_. All of the culture media were purchased from Sigma (St. Louis, MO, USA), and fetal bovine serum was purchased from Gibco (catalogue no. 16000-044; Grand Island, NY, USA).

### 4.3. Western Blotting

Samples from cell lysates or tissues lysates were separated by SDS-polyacrylamide gel electrophoresis, transferred onto polyvinylidene difluoride membranes, and incubated with primary antibodies, including: calpain-4 (1:1000; Abcam, Cambridge, MA, USA), and β-Actin (1:2000; Proteintech, Rosemont, IL, USA), followed by incubation with horseradish peroxidase (HRP)-conjugated secondary antibody (1:2000; Santa Cruz, Dallas, TX, USA). Protein expression was visualized by enhanced chemiluminescence assay.

### 4.4. Quantitative Real-Time PCR

Total RNA was isolated from frozen tissues and cell lines using TRIzol (Invitrogen, Carlsbad, CA, USA), according to the manufacturer’s instructions. RNA was used for the first-strand cDNA synthesis with a Takara RNA PCR Kit (Takara, Dalian, China). The quantitative RT-PCR assays were performed as described previously [29]. GAPDH was used as an internal control. The primers used were as follows: CAPN4 forward, 5′-CAGTTCGACACTGACCGATCAG-3′; CAPN4 reverse, 5′-CCCACTTTCATCTGAGTAGCGTC-3′; GAPDH forward, 5′-AAGGTCGGAGTCAACGGATTTG-3′; and GAPDH reverse, 5′-CCATGGGTGGAATCATATTGGAA-3′.

### 4.5. Immunohistochemistry

Standard protocols were used to prepare tissue microarray sections for immunohistochemistry. Then sections were blocked by UltraVision Hydrogen Peroxide Block (Thermo Scientific, Fremont, CA, USA) and UltraVision Protein Block (Thermo Scientific), followed by primary antibodies (CAPN4, 1:100) incubation. UltraVision Quanto Detection System horseradish peroxidase (HRP) Polymer (Thermo Scientific) and DAB Quanto (Thermo Scientific) were applied for staining, and hematoxylin was used for counterstaining. Immunohistochemical scoring was determined according to our previous report [30]. The staining intensity was scored as 0 to 3: 0, negative; 1, weak; 2, moderate; and 3, strong. Depending on the percentage of tumor cells that were positively stained, the heterogeneity of staining was scored as 0 to 4: 0, <5%; 1, 5%–25%; 2, 26%–50%; 3, 51%–75%; and 4, >75%. To obtain an IHC score that takes into account the IHC signal intensity and the frequency of positive cells, we generated a composite expression scores (CES) with full range from 0 to 12. CES is calculated from intensity and frequency measurements for immunostaining. CES = 4 (intensity score −1) + frequency score. According to receiver operating characteristic (ROC) analysis, CES of calpain-4 greater than or equal to 6 were considered to be high expression.

### 4.6. TCGA Dataset

These data are publically available from the Cancer Genome Atlas. All level-3 data were downloaded from the TCGA-STAD portal by using TCGA-Assembler software [31]. We selected a total of 386 samples of gastric cancer patients, available with both RNA-seq data and clinical features information for performing the correlation analysis. The mRNA expression in TCGA dataset was measured by RNA sequencing V2. The RSEM (RNA-Seq by Expectation-Maximization) counts were further normalized by TMM (trimmed mean of M value) method to estimate the relative RNA production levels using edgeR (empirical analysis of digital gene expression in R) software [32].

### 4.7. In Vitro Invasion Assays

Transwell matrigel invasion assays were performed in 24-well Hanging Inserts (8.0 μm PET (polyethylene terephthalate)) according to the manufacturer’s instructions (Millipore, Billerica, MA, USA). The bottom of transwell chamber was coated with Matrigel Basement Membrane Matrix (BD Biosciences, San Diego, CA, USA). The upper chamber was filled with 5 × 10^4^ cells in basic culture medium without serum. The lower chamber was filled with culture medium containing 20% FBS as a chemo-attractant. After the chambers were incubated for 24 h at 37 °C, non-invading cells on the upper side of the chamber were removed from the surface of the membrane by scrubbing, and invading cells on the lower surface of the membrane were fixed with 4% paraformaldehyde and stained with 0.1% crystal violet. The number of cells invading through the matrigel was counted in four randomly selected microscopic fields of each filter.

### 4.8. Statistical Analysis

Analysis was performed with SPSS software, version 22.0 (SPSS, Chicago, IL, USA) and R software (http://www.r-project.org/). The data are presented as the mean ± standard deviation, with at least three replicates used for each data point. Pearson’s chi-square test or Fisher’s exact test were used to compare the immunohistochemical variables and clinicopathologic characteristics. Survival was evaluated by the Kaplan-Meier survival curves, and the log-rank test was used to evaluate the differences between the groups. Univariate and multivariate survival analyses were performed using Cox regression. Nomogram was constructed by R software, version 3.0.2, with the “rms” package. A calibration plot was generated to examine the performance characteristics of the constructed nomogram. The predictive value of the parameters was assessed using the Akaike information criterion (AIC) and Harrell’s concordance index (*C*-index). Differences between two groups were analyzed with Student’s t test. Multiple comparisons were performed by one-way ANOVA test followed by dunnett’s post-hoc analysis (if the ANOVA turned out to be significant). Statistical significance was determined at the level of *p* < 0.05.

## 5. Conclusions

In summary, our data suggested that calpain-4 could be an independent prognosticator to establish the risk and prognosis of gastric cancer and to provide individualized therapeutic strategies in clinical practice. Future studies may focus on the molecular mechanisms of calpain-4 in gastric tumorigenesis and its role as a therapeutic target.

## Figures and Tables

**Table 1:** Correlation between calpain-4 expression and clinicopathological features of gastric cancer patients.

Calpain-4 Expression				
Variables	No.	Low	High	*p*-Value *
		No. (%)	No. (%)	
Gender				
Male	112	45 (40.2)	67 (59.8)	0.666
Female	62	27 (43.5)	35 (56.5)	
Age				
<60	84	32 (38.1)	52 (61.9)	0.395
≥60	90	40 (44.4)	50 (55.6)	
Tumor site				
Cardia	25	14 (56.0)	11 (44.0)	0.219
Body	38	13 (34.2)	25 (65.8)	
Antrum	111	45 (40.5)	66 (59.5)	
Lauren type				
Intestinal	109	47 (43.1)	62 (56.9)	0.808
Mixture	12	5 (41.7)	7 (58.3)	
Diffuse	53	20 (37.7)	33 (62.3)	
Differentiation				
Well/Moderately	34	17 (50.0)	17 (50.0)	0.255
Poorly	140	55 (39.3)	85 (60.7)	
Vessel invasion				
Positive	37	9 (24.3)	28 (75.7)	0.018
Negative	137	63 (46.0)	74 (54.0)	
T stage				
T1–T2	43	23 (53.5)	20 (46.5)	0.063
T3–T4	131	49 (37.4)	82 (62.6)	
Lymph node metastasis				
Positive	62	12 (19.4)	50 (80.6)	<0.001
Negative	112	60 (53.6)	52 (46.4)	
Distant metastasis				
M1	9	1 (11.1)	8 (88.9)	0.122
M0	165	71 (43.0)	94 (57.0)	
TNM stage				
I	34	21 (61.8)	13 (38.2)	0.006
II	39	19 (48.7)	20 (51.3)	
III	92	31 (33.7)	61 (66.3)	
IV	9	1 (11.1)	8 (88.9)	

*p* < 0.05 indicates that the differences have statistical significance. * Pearson chi-square tests.

**Table 2:** Univariate Cox regression analysis for overall survival of gastric cancer patients.

Variables	Univariate		
	HR	95% CI	*p*-Value
Gender			
Male vs. female	0.866	0.541–1.385	0.547
Age (years)			
≥60 vs. <60	1.436	0.923–2.235	0.108
Tumor site			
Cardia + body vs. antrum	1.440	0.905–2.290	0.124
Lauren type			
Diffuse + mixture vs. intestinal	1.029	0.642–1.651	0.905
Differentiation			
Poorly vs. well/moderately	1.264	0.725–2.200	0.409
Vessel invasion			
Positive vs. negative	1.861	1.052–3.293	0.033
T stage			
T3–T4 vs. T1–T2	2.923	1.791–4.772	<0.001
Lymph node metastasis			
Positive vs. negative	4.838	2.923–8.007	<0.001
Distant metastasis			
M1 vs. M0	14.93	4.203–53.01	<0.001
TNM stage			
III–IV vs. I–II	3.376	2.166–5.261	<0.001
Calpain-4 expression			
High vs. low	2.594	1.664–4.043	<0.001

CI, Confidence interval; HR, Hazard ratio; *p* < 0.05 was considered to be statistically significant.
